# A new method to control error rates in automated species identification with deep learning algorithms

**DOI:** 10.1038/s41598-020-67573-7

**Published:** 2020-07-03

**Authors:** Sébastien Villon, David Mouillot, Marc Chaumont, Gérard Subsol, Thomas Claverie, Sébastien Villéger

**Affiliations:** 1 MARBEC, Univ of Montpellier, CNRS, IRD, Ifremer, Montpellier, France; 2 Research-Team ICAR, LIRMM, Univ of Montpellier, CNRS, Montpellier, France; 3 University of Nîmes, Nîmes, France; 4 CUFR Mayotte, Dembeni, France; 5 Australian Research Council Centre of Excellence for Coral Reef Studies, James Cook University, Townsville, QLD 4811 Australia

**Keywords:** Ecology, Biodiversity, Community ecology, Conservation biology

## Abstract

Processing data from surveys using photos or videos remains a major bottleneck in ecology. Deep Learning Algorithms (DLAs) have been increasingly used to automatically identify organisms on images. However, despite recent advances, it remains difficult to control the error rate of such methods. Here, we proposed a new framework to control the error rate of DLAs. More precisely, for each species, a confidence threshold was automatically computed using a training dataset independent from the one used to train the DLAs. These species-specific thresholds were then used to post-process the outputs of the DLAs, assigning classification scores to each class for a given image including a new class called “unsure”. We applied this framework to a study case identifying 20 fish species from 13,232 underwater images on coral reefs. The overall rate of species misclassification decreased from 22% with the raw DLAs to 2.98% after post-processing using the thresholds defined to minimize the risk of misclassification. This new framework has the potential to unclog the bottleneck of information extraction from massive digital data while ensuring a high level of accuracy in biodiversity assessment.

## Introduction

In the context of accelerating human impacts on ecosystems^1^, the capacity to monitor biodiversity at large scale and high frequency is an urgent although challenging goal^2^. This urgency resonates with the ambition of international initiatives like the Group on Earth Observations Biodiversity Observation Network (GEO BON) and the call for monitoring Essential Biodiversity Variables (EBVs)^3,4^.

Remote sensors are rapidly transforming biodiversity monitoring in its widest sense from individuals^5^ to species and communities of species^6^. In the last decade, satellites^7,8^, drones^9, 10^, camera traps^6^, or underwater cameras^11,12^ have been extensively deployed to record pictures or videos of aquatic and terrestrial organisms. For instance, satellite data can be used to track whale shark movements^13^ or detect whales^14^ while photos from airborne or underwater vehicles can deliver accurate density estimations of vulnerable organisms like mammals or sharks^15,16^.

Such massive records are also used by citizen science programs with for example public tools like inaturalist.org which share pictures and associated metadata, or fishpix (https://fishpix.kahaku.go.jp) which offers the possibility to upload individual fish images that are then identified by experts at the species level.

However, processing photos or videos to identify organisms is a highly demanding task, especially in underwater environments, where some particular contexts add many difficulties (e.g., visual noise due to particles and small objects, complex 3D environment, color changing according to depth, etc.). For instance, identifying all fish individuals on videos may take up to 3 h of expert analysis per hour of video^17^. Under the avalanche of new videos and images to analyse, alternatives to fish identification by humans and trained experts must be found.

Recently, an effort to use machine learning methods^18,19^ and deep learning algorithms (DLAs) for ecological analysis have been made, thanks especially to computer-vision challenges on public databases of annotated photos or videos (e.g. for fish, Fish4Knowledge database (https://groups.inf.ed.ac.uk/f4k/) and Seaclef challenge (https://www.imageclef.org/lifeclef/2017/sea)).

The last generation of DLAs offer much promise for passing the bottleneck of image or video analysis through automated species identification^20–23^. DLAs, and particularly convolutional neural networks (CNNs), simultaneously combine the automatic definition of image descriptors and the optimization of a classifier based on these descriptors^24^. Even though DLAs usually have a high accuracy rate, they do not provide information on the confidence of the outputs. Hence, it remains difficult to identify and control potential misclassifications which limits their application.

Misclassification of images has two types of consequences for biodiversity monitoring. On one hand, if all individuals of a given species occurring in a given community are erroneously labelled as another species also occurring in the community, this species will be incorrectly listed as absent (false absence). The risk of missing present species because of misclassification is the highest for rare species, i.e. those with the lowest abundance in terms of the number of individuals per unit area. Yet missing these rare species can be critical for ecosystem heath assessment since some play important and unique roles like large parrotfishes on coral reefs^25^ while others are invasive like the lionfish in Eastern Mediterranean Sea^26^. In addition, since most species in a community are represented by a few individuals^27^, such misclassifications could significantly lead to the underestimation of species richness. The other error associated with misclassification is when an individual of a given species is mistaken for another species not present in the community (false presence). Such misclassifications could lead to an overestimation of the abundance or geographical range of a species as well as it could artificially increase species richness, unless a species is consistently mistaken for another. Since biodiversity monitoring should be as accurate as possible, automated identification of individuals on images should provide high correct classification rates (close to 100%) even if a subset of images has not been classified by the algorithm with sufficient confidence and must be identified by humans a posteriori.

Chow^28^ was the first to introduce the concept of risk for a classification algorithm. For instance, a clustering algorithm classifying an object placed in the center of a given cluster would present a low risk of misclassification, while classifying an object placed on the edge of a cluster would be highly risky. Chow^28^ proposed a classification framework, which contains *n* + 1 channels as outputs, *n* channels for the *n* classes considered and an additional channel called the “rejection” channel. When the risk of misclassification is too important, the algorithm rejects the classification.

Applied to machine learning, a first method consists in learning a rejection function during the training, in parallel to the classification learning^29–31^. Another method, called a meta-algorithm, uses two algorithms, one being a classifier, and the other one analyzing the classifier outputs, to distinguish predictions with a high risk of misclassification from those with a low risk^32^. A recent comparative study suggests that meta-algorithm-based methods are the most efficient^33^.

An extension of meta-algorithms to control the risk of misclassification is to calibrate models obtained through Machine Learning and Deep Learning algorithms. Machine Learning methods usually produce well-calibrated models for binary tasks^34^. The calibration consists of a matching between the score predicted by the machine-learning model and the real probability of true positives. While Deep Learning models produce more accurate classifications than other Machine learning models, these models are not well calibrated, and thus need a re-calibration to be used for real-world decisions^35^. Several propositions have been made to improve the calibration of Machine Learning models through the post-processing of outputs. The Platt scaling^36^, the Histogram binning^37^, the Isotonic Regression^38^ and the Bayesian Binning into Quantiles^39^ are mapping the model outputs to real accuracy probabilities. More recently, Temperature Scaling, an extension of the Platt Scaling, was used to calibrate Deep Learning models using a single parameter for all classes^35^. This parameter is used, instead of the traditional *softmax* function, to convert the vector output from the neural network into a real probability.

However, such calibration methods are based on a discretization of the Deep model outputs into bins. Many bins are not useful as they only contain a few outputs with low values, whereas many high values fall in the same bin and are thus not discriminated. Moreover, the choice of the number of bins is left to the user, and therefore is not optimized to the Deep model nor to a specific application^40^.

In this paper, we present a simple, yet efficient method that accounts for uncertainty in the classifier outputs. Unlike calibration methods, our approach is not changing algorithm outputs. Instead, we simply assess the behaviour of the model thanks to a validation dataset. We can then set-up a fine tuned threshold per class, allowing us to take into account that the model confidence can be highly variable between “easy” classes and “difficult” classes. Then, through the addition of a new class “unsure”, corresponding to predictions with scores lower than the predicted class threshold, we can control the coverage (total amount of images automatically identified) and misclassification rates. We applied this framework to classify 20 species of coral reef fishes in underwater images and assessed its efficiency for 3 real-case scenarios.

## Material and methods

### Data

We decided to build our own dataset instead of using existing datasets (e.g. Fish4Knowledge: https://groups.inf.ed.ac.uk/f4k/), to be in phase with quality of videos currently used by marine ecologists. We used 3 independent fish images datasets from the Mayotte Island (Western Indian Ocean) to train and test our CNN model and our post processing method. For the 3 datasets, we used fish images extracted from 175 underwater high-definition videos which lasted between 5 and 21 min for a total of 83 h. The videos were recorded in 1920 × 1,080 pixels with GoPro Hero 3 + black and Hero 4 + black. The videos were recorded between 2 and 30 m deep, with a broad range of luminosity, transparency, and benthic environment conditions on fringing and barrier reefs.

**Figure 1:**
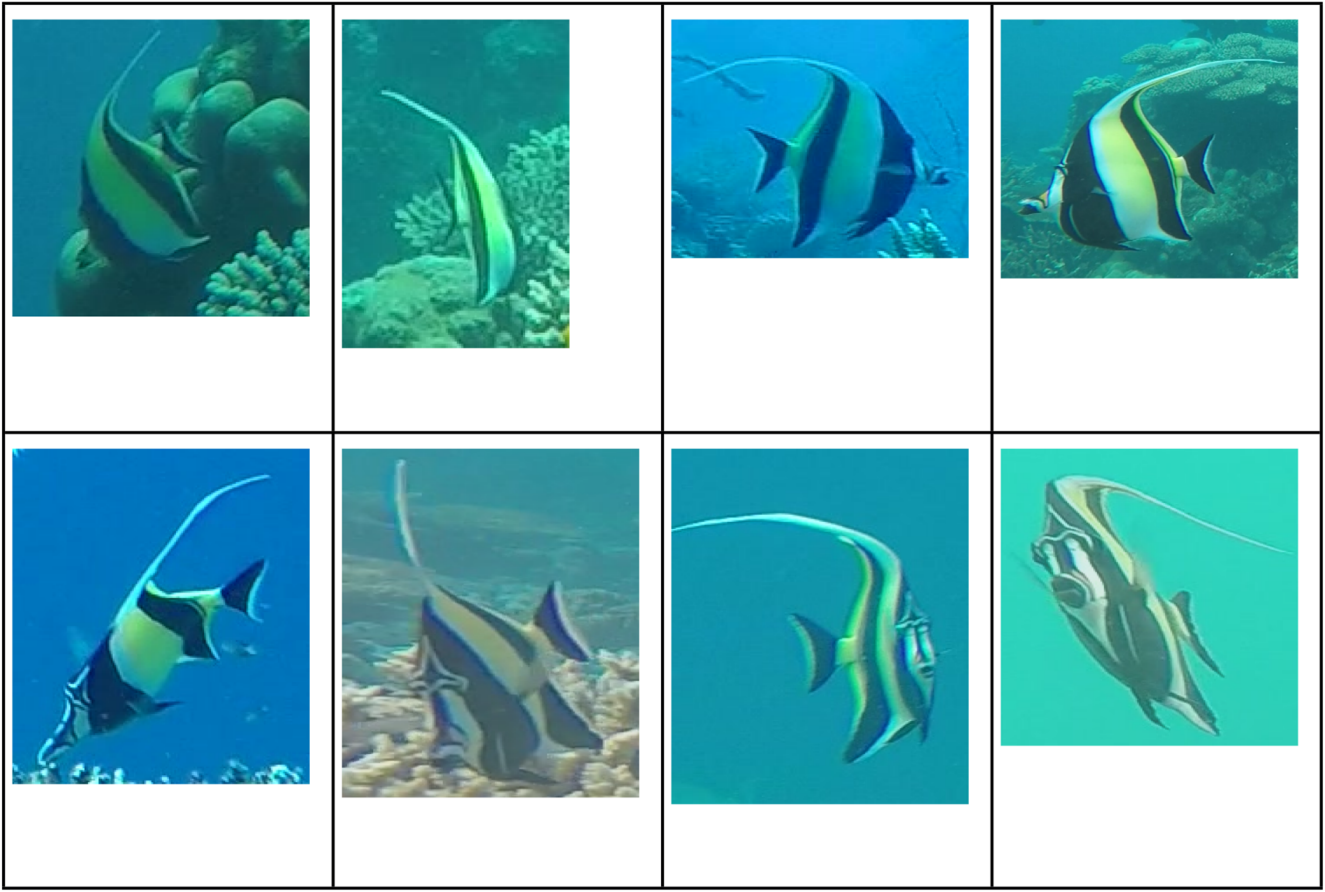
Diversity of individual images and their environment for the same fish species (Moorish idol, *Zanclus cornutus*).

We extracted 5 frames per second from these videos. Then, we cropped images to include only one fish individual with its associated habitat in the background. Thus, images of the same species differed in terms of size (number of pixels), colors, body orientation, and background (e.g. other fish, reef, blue background) (Fig. 1).

We used 130 videos for the training dataset, from which we extracted a total 69,169 images of 20 different fish species (Supplementary Fig. S1). We extracted between 1,134 and 7,345 images per species.

In order to improve our model, we used data augmentation^41^ on native biodiversity and ecosystem. Each “natural” image yielded 4 more images: 2 with increased contrast (120% and 140%) and 2 with decreased contrast (80% and 60%) (Supplementary Fig. S2). We then horizontally flipped all images to obtain our final training dataset (*T*0) composed of 691,690 images (Supplementary Table S1).

We then used two independent datasets made of different videos recorded on different days and on different sites than videos used to build the training dataset. The first dataset (*T*1) contained 6,320 images from 20 videos with at least 41 images per species, and the second (*T*2) contained 13,232 images from 25 videos with at least 55 images per species (Supplementary Table S1). We then used dataset *T*1 to tune the thresholds and *T*2 as the test dataset. This method ensures that our results are not biased by similar acquisition conditions between the training, tuning and testing dataset and hence that algorithm performance was evaluated using a realistic full cross-validation procedure.

### Building the convolutional neural network

Convolutional neural networks (CNNs) belong to the class of DLAs. For the case of species identification, the training phase is supervised, which means that the classes to identify are pre-defined by human experts while the parameters of the classifier are automatically optimized in order to accurately classify a “training” database^24^. CNNs are composed of neurons, which are organized in layers. Each neuron of a layer computes an operation on the input data and transfers the extracted information to the neurons of the next layer. The specificity of CNNs is to build a descriptor for the input image data and the classifier at the same time, ensuring they are both optimized for each other^42^. The neurons extracting the characteristics from the input data in order to build the descriptors are called convolutional neurons, as they apply convolutions, i.e. they modify the value of one pixel according to a linear weighted combination of the values of the neighbor pixels. In our case, each image used to train the CNN is coded as 3 matrices with numeric values describing the color component (R, G, B) of the pixel. The optimization of the parameters of the CNN is achieved during the training through a process called back-propagation. Back-propagation consists of automatically changing parameters of the CNN through the comparison between its output and the correct class of the training element to eventually improve the final classifications rate. Here we used a 100-layer CNN based on the TensorFlow^43^ implementation of ResNet^44^. The ResNet architecture achieved the best results on ImageNet Large Scale Visual Recognition Competition (ILSVRC) in 2015, considered as the most challenging image classification competition. It is still one of the best classification algorithms, while being very easy to use and implement.

All fish images extracted from the videos to build our datasets were resized to 64 × 64 pixels before being processed by the CNN. Our training procedure lasted 600,000 iterations; each iteration processed a batch of 16 images, which means that the 691,690 images of the training dataset were analyzed 14 times each by the network on average. We then stopped the training to prevent from overfitting^45^, as an over fit model is too restrictive and only able to classify images that were used during the training.

### Assigning a confidence score to the CNN outputs

The last layer of our architecture, as in most CNNs, is a “softmax” layer^44^. When input data passing through the network reaches this layer, a function is applied to convert the image descriptors into a list of *n* scores $S_{i}$, with $i = \left\{ {1,..,n} \right\},$ and *n* the number of learned classes (here the 20 different fish species), with the sum of all scores equal to 1. A high score means a “higher chance” for a given image to belong to the predicted class. However, a CNN often outputs a class with a very high score (more than 0.9) even in case of misclassification. To prevent misclassifications, the classifier should thus be able to add a risk or a confidence criterion to its outputs.

### Assessing the risk of misclassification by the CNN

For a given input image, a CNN returns a predicted class, in our case a fish species. As seen in the previous section, the CNN outputs a decision based on the score, without any information on the risk of making an error (i.e. a misclassification). Following De Stefano et al.^32^, we thus propose to apply a post-processing step on the CNN outputs in order to accept or reject its classification decision. The hypothesis is that the higher the similarity between an unknown image and the images used for the training, the stronger the activation in the CNN during the classification process (i.e. the higher the score is), and thus, the more robust the classification is.

For this method, the learning protocol is thus made of two consecutive steps performed on 2 independent training datasets.

**Figure 2:**
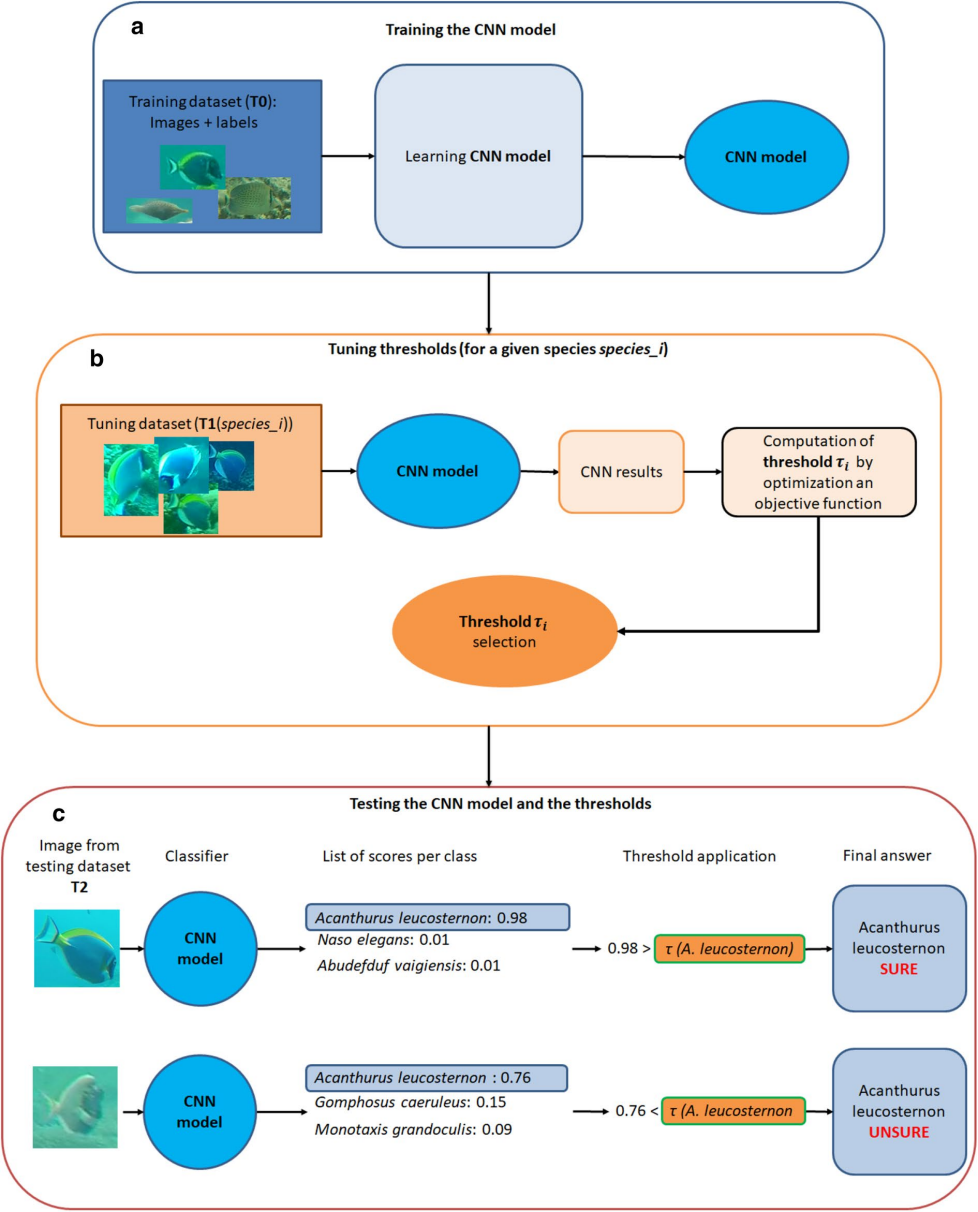
Overview of the 3 parts of our framework: 2 consecutive steps for the learning phase, followed by the applicative testing step. (**a**) We trained a CNN model with a training dataset (*T*0) composed of images and a label for each image, in our case, the species corresponding to each fish individual. (**b**) Then, for each species *i*, we processed an independent dataset *T*1, with our model. For each image, we obtained the species *j* attributed by the CNN to the image and a classification score $S_{j}$. We have the ground truth and the result of the classification (correct/incorrect), so we can define a threshold according to the user goal. This goal is a trade-off between the accuracy of the result and the proportion of images fully processed. (**c**) We then used this threshold to post-process outputs of the CNN model. More precisely, for a given image, the classifier of the CNN returns a score for each class (here for each fish species). The most likely class $C\left( X \right)$ for this image is the one with the highest score $S\left( X \right)$ We then compared this highest score $S\left( X \right)$ with the computed confidence threshold for this species *(*$\tau_{C\left( X \right) }$*)* obtained in the second phase. If the score was lower than the computed threshold that is $S\left( x \right) > \tau_{C\left( X \right) }$, then the input image was classified as “unsure”. Otherwise, we kept the CNN classification.

- In the first phase, a classification model is built by training a CNN on a given database *T*0 (Fig. 2a)
- Then, the second phase consists of tuning a risk threshold $\tau_{i }$ specific to each class (i.e. each species in our case), noted *i,* with $i \in \left\{ {1,...,n} \right\}$, using a second and independent database noted *T1* (Fig. 2b).

**Figure 3:**
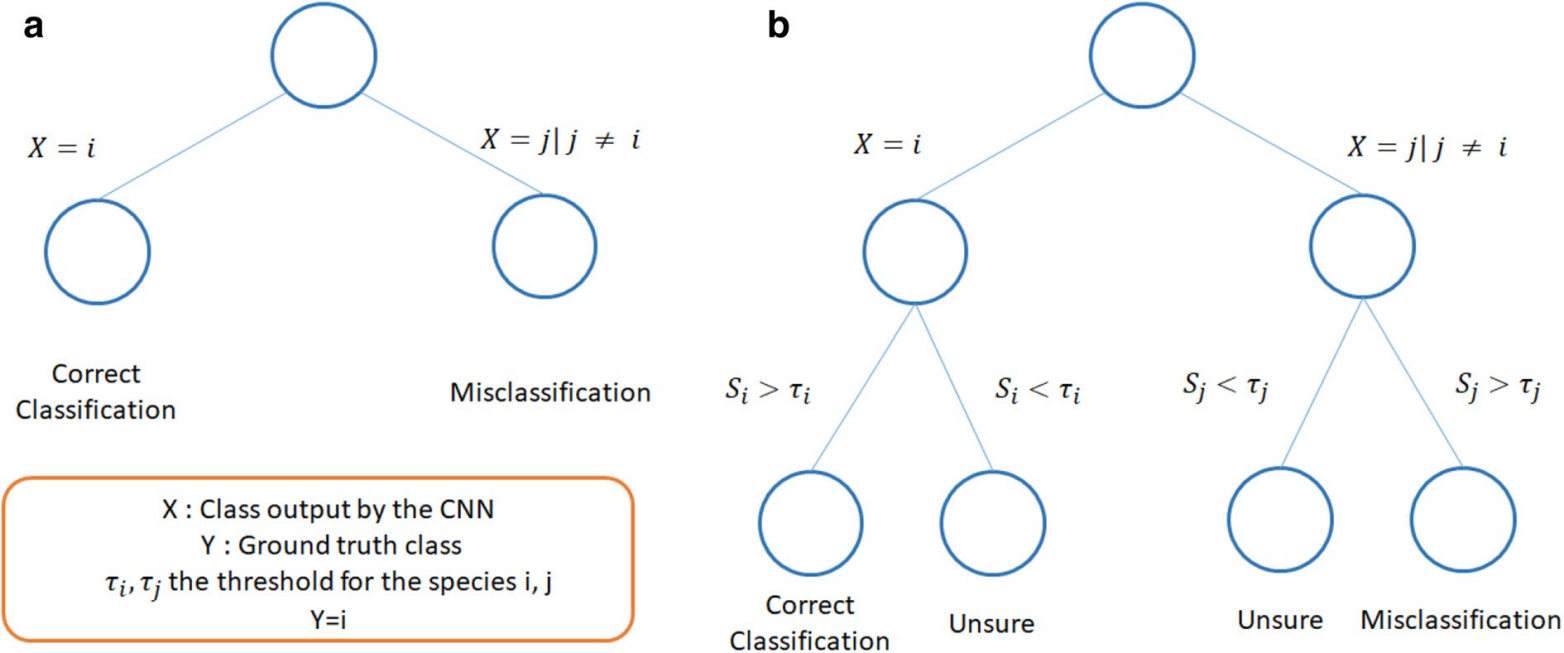
Impact of the post-processing framework on classification of images for a given species and a given threshold. Usually, the classification of an image of class *i* can either be correct, if the model classifies it as *i,* or wrong, if the classifier classifies it as *j* with *j* $\ne$*i* (**a**). We propose a post processing to set a confidence threshold for each class to obtain 3 types of results, correct, misclassified, and unsure (**b**). The goal is then to transform as many misclassifications as possible as “Unsure”, while preventing to transform too many correct classifications “Unsure”.

In terms of classification, it means we transform the 2 classification options (correct, wrong) in 3 options (Fig. 3) by applying Eqs. (15, 16).

### Computing the confidence thresholds

After the phase 1 (model training phase), for an image $X$ of the threshold tuning dataset processed by the classifier, we obtain an output $\left\{ {C\left( X \right),S\left( X \right)} \right\}$, where $C\left( X \right)$ is the class (i.e. species, belonging to the trained set of species) with the highest classification score $S\left( X \right).$ For this image, we know the ground truth $Y$ in $\left\{ {1,..,n} \right\}$ belonging to the same set of species classes.

So with $C\left( X \right)$ being the output class, $Y$ the ground truth class, and $\# \left( . \right)$ the enumeration function, the standard definition for Correctly Classified images (or true positives) rate of a class $i$ is:

1 $$CC_{i} = \frac{{\# \left( {C\left( X \right) = i ~AND~ Y = i} \right)}}{\# Y = i}$$

We can also write the standard definition of Misclassified images rate (or false negatives) of a class $i$ as:

2 $$MC_{i} = \frac{{\# \left( {C\left( X \right) \ne i~ AND~ Y = i} \right)}}{\# Y = i}$$

Then, we can extend the Correct Classification rate (CC) and Misclassification (MC) rate of a species $i$ by introducing the thresholds $\tau_{i }$ and by adding the Unsure Classification (UC) rate:

3 $$CC_{i} \left( {\tau_{i } } \right) = \frac{{\# (\left( {C\left( X \right) = i} \right) AND (S\left( X \right) > \tau_{i } )) AND \left( {Y = i} \right)}}{{\# \left( {Y = i} \right)}}$$

4 $$MC_{i} \left( {\tau_{i } } \right) = \frac{{\# \left( {\left( {C\left( X \right) \ne i) AND (S\left( X \right) > \tau_{i } } \right)} \right) AND \left( {Y = i} \right)}}{{\# \left( {Y = i} \right)}}$$

5 $$UC_{i} \left( {\tau_{i } } \right) = \frac{{\# \left( {\left( {C\left( X \right) = i} \right) OR \left( {C\left( X \right) \ne i } \right)} \right) AND (S\left( X \right) < \tau_{i } ) }}{{\# \left( {Y = i} \right)}}$$

For each species we have:

6 $$CC_{i} \left( \tau \right) + MC_{i} \left( \tau \right) + UC_{i} \left( \tau \right) = 1$$

We can also note that the standard coverage definition (COV, the rate of images for which a classification is given) of a species $i$ can be extend with the introduction of thresholds as threshold $\tau$ as:

7 $$COV_{i} \left( \tau \right) = CC_{i} \left( \tau \right) + MC_{i} \left( \tau \right)$$

The question is now to select “optimal” thresholds $\left\{ {\tau_{i } } \right\}_{i = 1}^{i = n}$ based on the dataset T1. This is not straightforward as is it up to user specific objective, such as minimizing MC, maximizing CC, minimizing UC… In the following, we analyze three different goals corresponding to some standard protocols in marine ecology:

- The first goal *G1* consists of keeping the best correct classification rate while reducing the misclassification error rate. For this, we used two steps. First, we identified the threshold(s) $\tau$ which maximizes $CC_{i} \left( \tau \right)$. Since several thresholds could reach this maximum, we get a set of threshold(s) $Se_{g1}$. Then, we selected the threshold with the lower $MC_{i} \left( \tau \right)$. This can be mathematically written as:
  8 $$Se_{g1} = \mathop {\text{arg max}}\limits_{\tau } CC_{i} \left( \tau \right)$$
  9 $$\tau_{i } = \mathop {\text{arg min}}\limits_{{\tau ^{\prime} in Se_{g1} }} MC_{i} \left( {\tau ^{\prime}} \right)$$
- The second goal *G2* consists in constraining the misclassification error rate to an upper bound of 5% while maximizing the correct classification rate. Reaching this goal requires to first find $Se_{g2}$ the set of threshold(s) such as $MC_{i} \left( \tau \right)$ < 5%. If there is none, we considered $Se_{g2}$ as the set of threshold(s), which minimize $MC_{i}$. Then we defined the optimal threshold $\tau_{i }$ by choosing the one in $Se_{g2}$ which maximizes $CC_{i}$:
  10 $$Se_{g2} { } = \tau /MC_{i} \left( \tau \right) < 5\%$$
  11 $$if\, Se_{g1} = \emptyset\, then\, Se_{g2} = \mathop {\text{arg min}}\limits_{\tau } MC_{i} \left( \tau \right)$$
  12 $$\tau_{i } = \mathop {\text{arg max}}\limits_{{\tau ^{\prime} in Se_{g2} }} CC_{i} \left( {\tau ^{\prime}} \right)$$
- The third goal G3 consists of keeping the lowest misclassification rate while raising the correct classification error rate (implying a lower coverage). First, we defined $Se_{g3}$ as the set of threshold(s) $\tau$ that minimizes $MC_{i} \left( \tau \right)$. If there were several thresholds with the same minimal value, we chose $\tau_{i }$ as the one which maximizes $CC_{i}$:
  13 $$Se_{g3} = \mathop {\text{arg min}}\limits_{\tau } MC_{i} \left( \tau \right)$$
  14 $$\tau_{i } = \mathop {\text{arg max}}\limits_{{\tau ^{\prime} in Se_{g3} }} CC_{i} \left( {\tau ^{\prime}} \right)$$

For a given image X in the test dataset, the classification and post-process is sequential as follows (Fig. 2c):

- First, the image is given to the CNN, which outputs a list of scores, including $S\left( X \right)$ the highest score obtained by a class.
- Second, for the class $$C\left( X \right)$$ (i.e. the class with the highest classification score), the post-processing step estimates the risk of classifying the image as belonging to the class $$C\left( X \right)$$. If $$\left( X \right) <$$$$\tau_{j }$$, the prediction is changed to “Unsure”, otherwise, it is confirmed as the class *j* (Fig. 2c).

The misclassification rate for a species $Y = i$ after post-processing thus equals:

15 $$MC^{\prime}_{i} = \frac{{\# \left( {\left( {C\left( X \right) \ne Y} \right) AND \left( {S\left( X \right) > \tau_{j} } \right)} \right) AND \left( {Y = i} \right)}}{{\# \left( {Y = i} \right)}}$$

and the unsure classification rate equals:

16 $$UC^{\prime}_{i} = \frac{{\# \left( {\left( {C\left( X \right) = j} \right) AND \left( {S\left( X \right) < \tau_{j} } \right)} \right) AND \left( {Y = i} \right)}}{{\# \left( {Y = i} \right)}}$$

First, to assess the effectiveness of our framework, we processed all the images contained in *T*2 through the DL algorithm, without post processing (threshold tuning + threshold application).

Second, we assessed whether a unique threshold for all the classes was sufficient to separate correct classifications from misclassifications for all species. For this test, we computed the distribution of correct classifications and misclassifications over scores for each species. During this study, we multiplied the softmax scores, which ranged from 0 to 1, by 100, for an easier reading.

Then, to study the impact of the post-processing method in an hypothetical ideal condition, we selected the thresholds based on the dataset *T*2 and we applied them to the same dataset *T*2. For this experiment and the following, we also measured both the Correct Classification rate and the Accuracy, defined for a species $i$ as

$$Accuracy_{i} = \frac{{\# \left( {\left( {C\left( X \right) = i} \right) AND \left( {S\left( X \right) > \tau_{i} } \right)} \right) AND \left( {Y = i} \right)}}{{\# \left( {\left( {C\left( X \right) = i} \right) AND \left( {S\left( X \right) > \tau_{i} } \right)} \right)}}$$

The accuracy varies from 0 to 1, and increases when the number of false positives decreases and the number of true positives increases. Meanwhile, the CC rate varies from 0 to 100, and increases when the number of false negatives decreases and the number of true positives increases.

Finally, to ensure that the post-processing method was relevant for any real-life application, i.e. that thresholds are defined and tested on independent datasets, we used the dataset *T*1 for the threshold-setting phase and the dataset *T*2 for the testing phase. To assess the robustness of our method, we repeated the same experiment while switching the roles of *T*1 and *T*2. Note that we limited our experiments to the use of T1 and T2, but that it could be interesting in further work to assess the robustness of this method with datasets composed of less data.

## Results

### Results of the CNN model classification

The mean rate of correct classification of fish images in*T*2 by the raw CNN was of 78.0%, with rates of correct classifications per species ranging from 54.4% to 99.1% (sd = 15.16) (Table 1). These results are the baseline for our following experiments.

**Table 1:** Output of the deep learning classifier without post-processing. Percentages of correct classifications are shown for the 20 fish species. Each line shows the species name, the correct classification rate of images of this species present in the dataset *T2*, the softmax score above which we have 95% of the correct classification (noted sq0.05), and the percentage of Misclassified images with score equal or above sq0.05.

Species	Test dataset *T*2 (% of correct classifications)	Softmax score for the 0.05 quantile of correct classification (sq0.05)	% of Misclassification for sq0.05
*Chaetodon trifasciatus*	87.80	99.91	20
*Chaetodon trifascialis*	90.00	99.98	11.11
*Naso brevirostris*	54.14	99.92	29.91
*Chaetodon guttatissimus*	85.50	99.82	10.77
*Thalassoma hardwicke*	90.90	99.92	0
*Pomacentrus sulfureus*	90.14	99.88	0
*Oxymonacanthus longirostris*	96.43	99.98	0
*Monotaxis grandoculis*	57.10	98.78	34.1
*Zebrasoma scopas*	63.04	96.78	19.92
*Abudefduf vaigiensis*	99.07	99.99	0
*Amblyglyphidodon indicus*	58.78	92.85	22.04
*Acanthurus lineatus*	59.72	99.98	16.38
*Chromis ternatensis*	59.61	86.74	26.98
*Chromis opercularis*	61.29	99.00	16.67
*Gomphosus caeruleus*	75.72	99.84	33.33
*Acanthurus leucosternon*	86.15	99.94	16.65
*Halichoeres hortulanus*	82.93	99.96	16.33
*Naso elegans*	93.24	99.78	6.46
*Chaetodon auriga*	87.05	99.98	10.77
*Zanclus cornutus*	81.36	99.68	9.1
Mean	78.00	98.64	17.49
Standard Deviation	15.16	3.27	10.84

**Figure 4:**
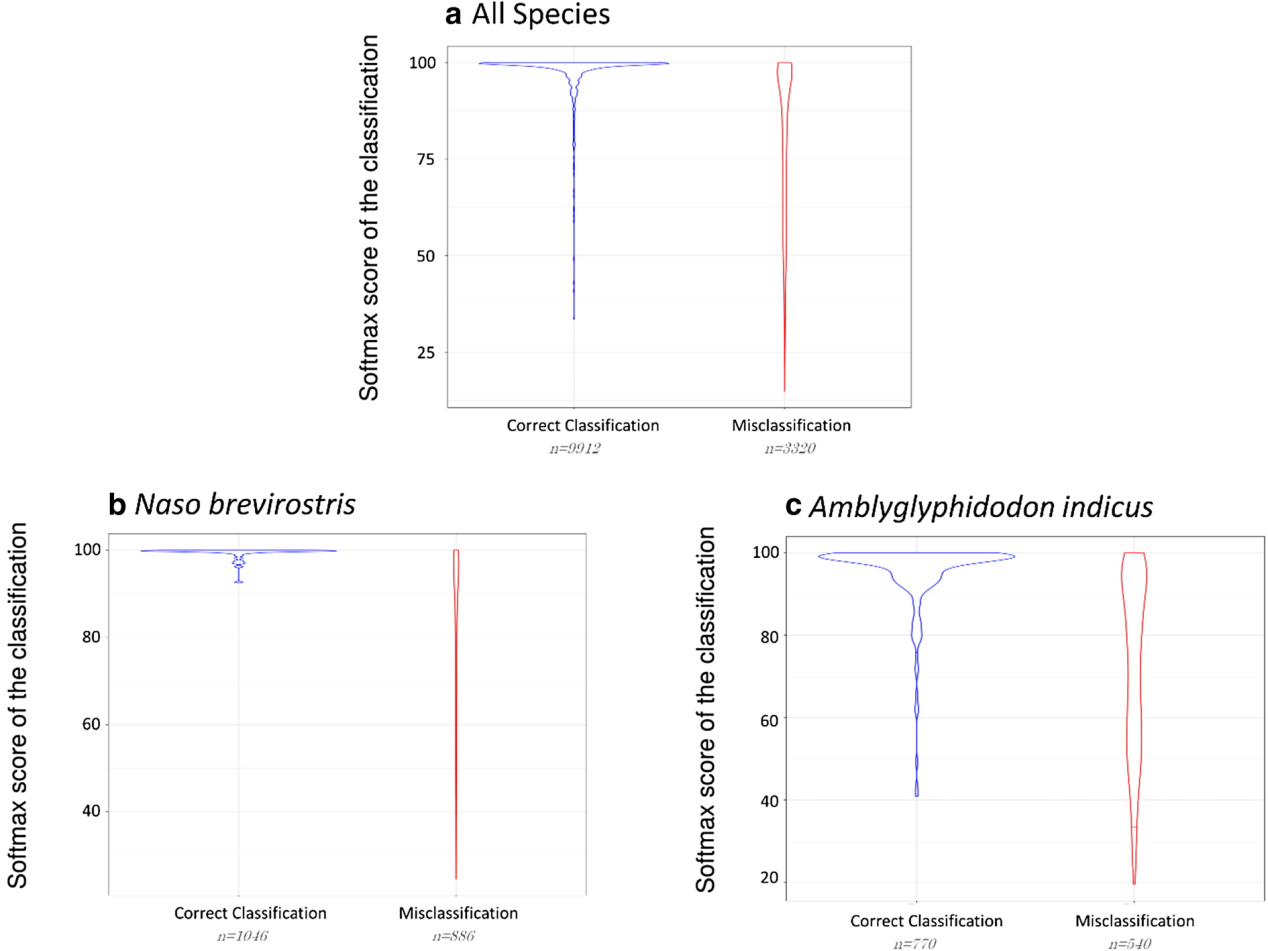
Distribution of correct classifications and misclassifications of fish images with respect to the score from the CNN model. We plotted the results for all species (**a**), and for 2 species, the Brown unicornfish (*Naso brevirostris*) (**b**) and the Maldives damselfish (*Amblyglyphidodon indicus*) (**c**). We also plotted the 5% bottom line for each type of classification. We used violin plots for the visualisation. Violin plot are histograms with inverted axis allowing a graphical visualisation of a distribution, with the number of individuals on the Y axis and their value on X axis. The borders of the shapes show the number of individuals while the dots show the local density”^46^.

Images obtained softmax scores between 41 and 100 with 80% of images classified with a score between 60 and 100 (Fig. 4a). The distribution of correct classifications and misclassifications among scores was highly variable among species (Fig. 4b,c, Table 1).

### Benchmark of the threshold fine-tuning method

**Figure 5:**
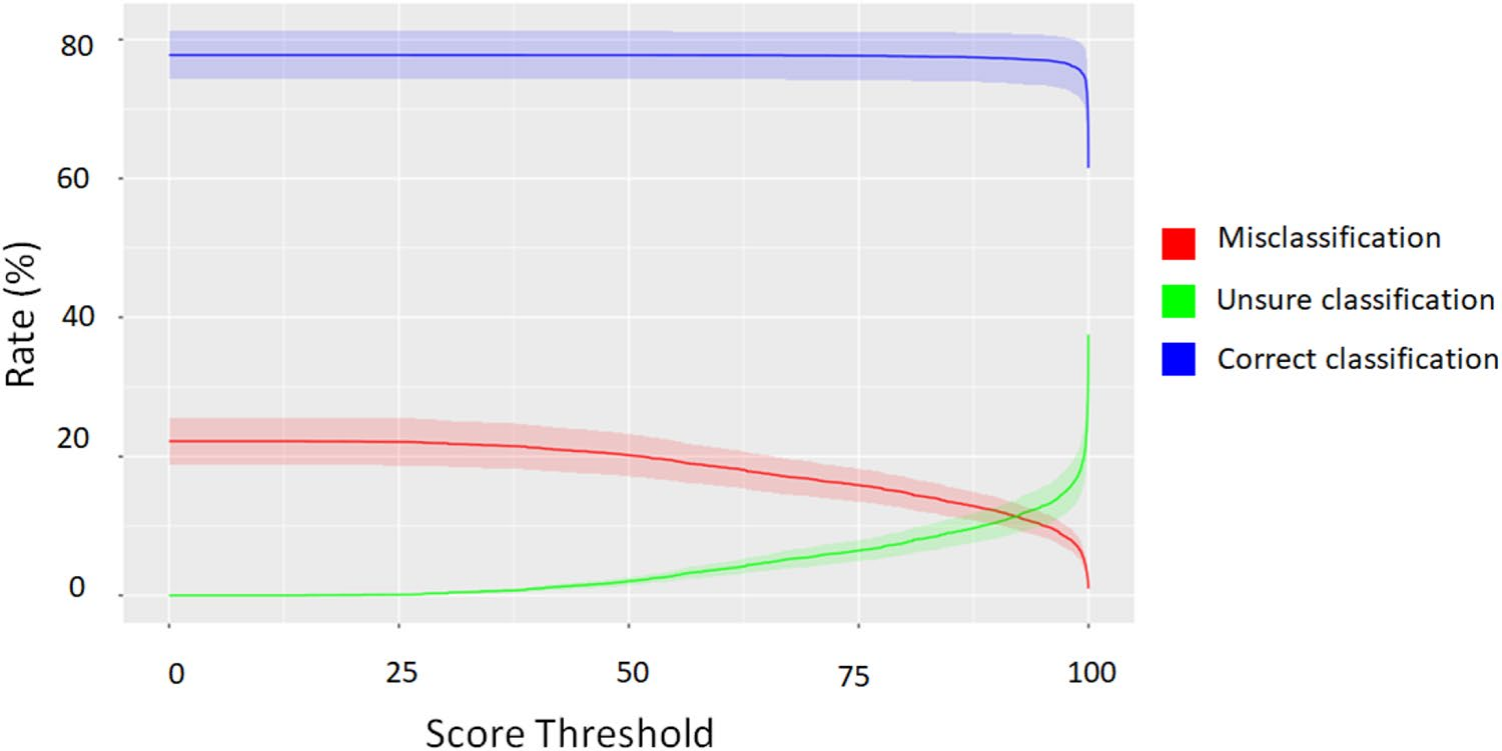
Average distribution of correct, wrong, and unsure classifications for all species along a gradient of confidence threshold score.

For each species *i*, we computed $CC_{i}$, $MC_{i}$, $UC_{i}$ values while varying the threshold. We computed and applied the thresholds on *T2*, according to Eqs. 8–16. As the score varied from 0 to 99.9, the misclassification rate decreased to 0.9% (Fig.  5). This decrease was mainly compensated by the increasing rate of unsure classifications between 0 and 99.9 of classification scores.

Indeed, the rate of correct classifications experienced little variation along this distribution of threshold scores, remaining between 74 and 78% for threshold scores between 0 and 99.8 and decreasing to 61% for threshold scores > 99.8. However, correct, wrong, and unsure classification rates were highly variable among species (Supplementary Table S2).

**Figure 6:**
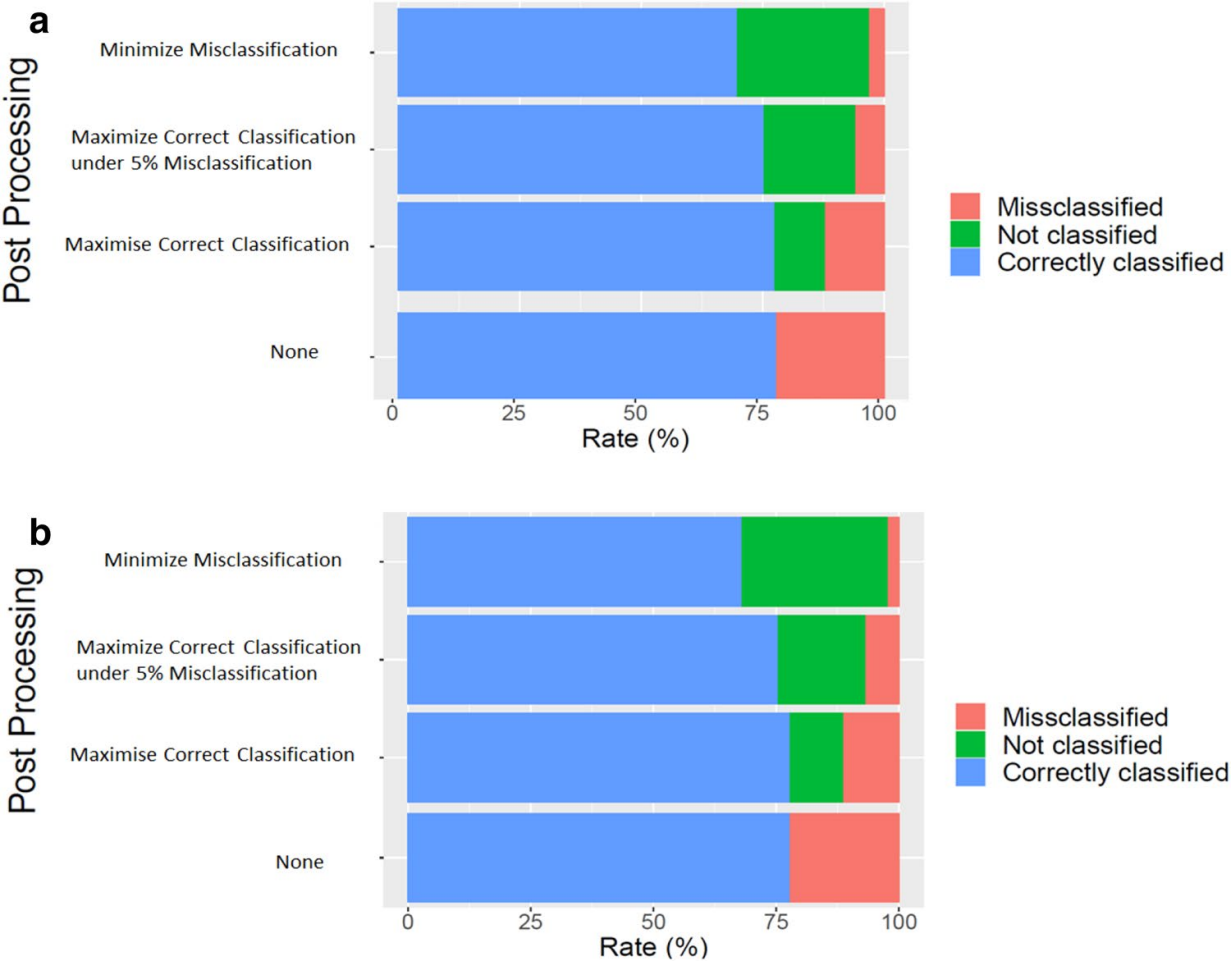
Benchmark scenario and cross–validation classification rates. We compare results obtained by tuning the thresholds on *T*2 and using *T2* as a testing set (**a**) and real-life scenario obtained by tuning the thresholds on *T*1 and using *T2* as a testing set (**b**). For sub-figure: From top to bottom, rates of correct classifications, misclassifications, and unsure classifications for each post-processing: (1) Goal 1: Minimizing misclassification with $CC_{i} = \max CC_{i}$, (2) Goal 2: maximizing correct classifications under the constraint of having less than 5% of misclassifications, (3) Goal 3: maximizing correct classification with $MC_{i} = \min MC_{i}$, (4) No post-Processing.

For the first goal G1, we defined the thresholds (one per species) to minimize the misclassification with $CC_{i} = \max CC_{i}$. We obtained a mean rate of 78% (standard deviation = 15.15%) of correct classifications, 10.81% (s.d = 8.15%) of unsure classifications, and 11.19% (s.d = 9.58%) of misclassifications (Fig. 6a).

For the second goal G2, we maximized the correct classifications while constraining the misclassification error rate to an upper bound of 5% (if possible). We obtained a rate of 75.47% (s.d = 17.83%) of correct classifications, 17.88% (s.d = 14.22%) of unsure classifications, and 6.66% (s.d = 6.44%) of misclassifications.

For the third goal G3, we maximized the number of correct classifications with $MC_{i} = \min MC_{i}$. We obtained a rate of 68.21% (s.d = 22.41%) of correct classifications, 29.71% (s.d = 22.14%) of unsure classifications, and 2.07% (s.d = 3.20%) of misclassifications, on average. Compared to the first goal, we decreased the rate of correct classifications by 8.9% and the rate of misclassifications by 2.6% (Supplementary Table S4).

The accuracy of the goals G1, G2, and G3 were, on average, higher than the raw accuracy (0.53) with respectively 0.72, 0.89 and 0.94 (Table 2).

**Table 2:** Accuracy of the models without post-processing, and with post processing according to our goals, with thresholds tuned and applied on *T2*.

Species	Raw Accuracy	G1 Accuracy	G2 Accuracy	G3 Accuracy
*Abudefduf vaigiensis*	0.51	0.65	0.9	0.97
*Acanthurus leucosternon*	0.61	0.69	0.87	0.96
*Acanthurus lineatus*	0.87	0.91	0.97	0.97
*Amblyglyphidodon indicus*	0.08	0.74	0.94	0.98
*Chaetodon auriga*	0.95	0.99	1	1
*Chaetodon guttatissimus*	0.16	0.84	0.95	0.98
*Chaetodon trifascialis*	0.97	0.87	0.95	0.96
*Chaetodon trifasciatus*	0.56	0.62	0.79	0.97
*Chromis opercularis*	0.68	0.8	0.96	1
*Chromis ternatensis*	0.01	0.44	0.79	0.9
*Gomphosus caeruleus*	0.24	0.31	0.54	0.72
*Halichoeres hortulanus*	0.51	0.59	0.8	0.93
*Monotaxis grandoculis*	0.77	0.81	0.96	0.99
*Naso brevirostris*	0.02	0.9	0.96	1
*Naso elegans*	0.89	0.92	0.97	0.97
*Oxymonacanthus longirostris*	0.36	0.46	0.89	0.85
*Pomacentrus sulfureus*	0.52	0.7	0.91	0.95
*Thalassoma hardwicke*	0.78	0.85	0.93	0.95
*Zanclus cornutus*	0.55	0.68	0.87	1
*Zebrasoma scopas*	0.61	0.7	0.81	0.81
Mean	0.53	0.72	0.89	0.94
Standard deviation	0.30	0.18	0.10	0.07

Each line shows the result for a species, with: the species name, the accuracy of the model without post processing, and the accuracy of the model with post processing according to the 3 goals defined earlier.

The thresholds showed higher variations among species for G1, with values ranging from 33.46 to 99.97, than for G3 for which values ranged from 99.86 to 99.98 among the 20 species (Supplementary Tables S2, S3).

### Application of the method

For a real cross-validation experiment, thresholds were set using *T*2 and then applied on *T*1*.* The correct, wrong and unsure classification rates were very close (difference < 2.6%) to those obtained with the benchmark situation (Supplementary Table S5).

The proposed post-processing was able to decrease the misclassification rate by at least 10.05%, for all goals, and 19.02% at most compared to the raw output of the Deep Learning model (Fig. 6b). The accuracy followed the same tendency, with an average accuracy for G1, G2 and G3 respectively equal to 0.74, 0.81 and 0.92 (Table 3).

**Table 3:** Accuracy of the model without post-processing, and with post processing according to our goals, on the cross-validation, with thresholds tuned on *T1* and applied on *T2*.

Species	Raw Accuracy	G1 Accuracy	G2 Accuracy	G3 Accuracy
*Abudefduf vaigiensis*	0.51	0.61	0.92	0.97
*Acanthurus leucosternon*	0.61	0.7	0.92	0.94
*Acanthurus lineatus*	0.87	0.91	0.95	0.97
*Amblyglyphidodon indicus*	0.08	0.72	0.97	0.97
*Chaetodon auriga*	0.95	0.99	0.95	1
*Chaetodon guttatissimus*	0.16	0.88	0.72	0.96
*Chaetodon trifascialis*	0.97	0.9	0.96	0.98
*Chaetodon trifasciatus*	0.56	0.62	0.43	0.85
*Chromis opercularis*	0.68	0.83	0.03	1
*Chromis ternatensis*	0.01	0.47	0.97	0.87
*Gomphosus caeruleus*	0.24	0.31	0.89	0.75
*Halichoeres hortulanus*	0.51	0.57	1	0.9
*Monotaxis grandoculis*	0.77	0.82	0.99	0.98
*Naso brevirostris*	0.02	0.92	0.89	1
*Naso elegans*	0.89	0.91	0.99	0.97
*Oxymonacanthus longirostris*	0.36	0.46	0.72	0.8
*Pomacentrus sulfureus*	0.52	0.92	0.71	0.91
*Thalassoma hardwicke*	0.78	0.94	0.77	0.94
*Zanclus cornutus*	0.55	0.64	0.4	0.98
*Zebrasoma scopas*	0.61	0.66	0.99	0.8
Average	0.53	0.74	0.81	0.93
Standard deviation	0.30	0.19	0.25	0.07

Each line shows the result for a species, with: the species name, the accuracy of the model without post processing, and the accuracy of the model with post processing according to the 3 goals defined earlier.

Finally, we also performed the same experiment while switching *T1* and *T2* roles (Supplementary Tables S6, S7, S8). For each goal, the unsure classification rate was higher after the switch (+ 3.8% for G1, + 4.4% for G2, and + 8.9% for G3), implying lower scores were obtained in both correct classification (− 3.5%, − 5%, − 7.3%) and misclassification, with the exception of the 2nd goal (-0.2%, + 0.6%, − 1.6%).

## Discussion

Biodiversity monitoring is experiencing a revolution with the emergence of new sensors (light, noise, image, environmental DNA) that generate massive datasets and require powerful and accurate treatment tools. Indeed, species misclassifications must be controlled and limited to avoid false negatives or absences i.e., missing species that are actually present and false positives or presences i.e., detecting species that are actually absent.

In this paper, we demonstrated that the risk of misclassification by CNN algorithms can be measured and controlled in a post-processing step to provide more accurate identification of species on pictures. Such post-processing can be applied with any classifier as long as the output is a vector of scores. Reducing the misclassification rate is at the detriment of the correct classification rate and increases “unsure” classifications, which implies a low coverage and a greater human effort needed to identify unclassified individuals. Hence, there is a trade-off between a more secure (less misclassifications) or a more automatic (more classifications) method so species thresholds can be set according to the goal or priority of the study or the availability and time of experts. Here we define three main goals which represent archetypal study cases. The first goal, maximizing the correct classification rate but not limiting misclassifications, can be applied when avoiding false negatives is more important than detecting false positives. This can be the case for monitoring invasive species, since the priority is to detect the first occurrence of such invasive individuals with potential deleterious consequences on native biodiversity and ecosystem functioning^47^ particularly on islands^48,49^. For instance, the Indo-Pacific predator lionfish (*Pterois volitans* and *P. miles*) has invaded most reefs of the Western Atlantic and depleted many native prey populations, and are starting to spread in the Eastern Mediterranean Sea^12^. To better anticipate the impact of such species, ecosystem mangers needs to be aware of the first occurrence on reefs and can thus accept having “false alarms”. The same constrains applies for detection particular or emblematic individuals, like Whale Sharks, through photo-identification^50^ where the primary goal is to avoid missing an occurrence. In both ecological cases, experts will eventually validate the few false positive identifications of targeted organisms by the algorithm to discard them.

The second goal, maximizing the correct classification rate while limiting misclassifications at 5% maximum per species, can be applied when avoiding false negatives and false positives are both important. This is the trade-off scenario that requires the least human effort and that can process massive datasets with few errors. It can be recommended to analyze long videos (> 2 h) for monitoring biodiversity metrics that are weakly influenced by undetected species (rare or classified as “unsure”), like the assessment of taxonomic or functional diversity^25^, and that can feed initiatives like the Group on Earth Observations Biodiversity Observation Network (GEO BON) and provide robust estimates of Essential Biodiversity Variables (EBVs)^3,4^.

The third goal, minimizing the misclassification rate, can be applied when detecting false positives is more problematic than avoiding false negatives, which creates many “unsure” classifications. This can be the case when priority is to accurately analyze a relatively small dataset with the support of many experts who can help to identify species on potentially a high number of “unsure” images. For instance, assessing abundance of all species within a given area to explain ecosystem functioning (e.g.^51^) or to monitor changes in species relative abundances (e.g.^52^) requires a minimum number of misclassifications.

Whatever the goal, our framework is highly flexible and can be adapted by tuning the species thresholds regulating the trade-off between classification robustness and coverage in an attempt to monitor biodiversity through big datasets where species are unidentified. To unclog the bottleneck of information extraction about organism forms, behaviors and sounds from massive digital data, machine learning algorithms, and particularly the last generation of deep learning algorithms, offer immense promises. Here we propose to help the users to control their error rates in ecology. This is a valuable addition to the ecologist’s toolkit towards a routine and robust analysis of big data and real-time biodiversity monitoring from remote sensors. With this control of error rate in the hands of users, Deep Learning Algorithms can be used for real applications, with acceptable and controlled error rates, lower than any state of the art fully automatic process, while fixing the effort by human experts to correct algorithm mistakes.

## Supplementary information

Supplementary file1 (DOCX 3278 kb)
