# Author Correction: A new method to control error rates in automated species identification with deep learning algorithms

**DOI:** 10.1038/s41598-020-72312-z

**Published:** 2020-09-04

**Authors:** Sébastien Villon, David Mouillot, Marc Chaumont, Gérard Subsol, Thomas Claverie, Sébastien Villéger

**Affiliations:** 1 MARBEC, Univ of Montpellier, CNRS, IRD, Ifremer, Montpellier, France; 2 Research-Team ICAR, LIRMM, Univ of Montpellier, CNRS, Montpellier, France; 3 University of Nîmes, Nîmes, France; 4 CUFR Mayotte, Dembeni, France; 5 Australian Research Council Centre of Excellence for Coral Reef Studies, James Cook University, Townsville, QLD 4811 Australia

Correction to: *Scientific Reports* 10.1038/s41598-020-67573-7, published online 03 July 2020

The information in the Article is incomplete. The paper does not discuss the related works of Jessica Y. Luo and colleagues, and Robin Faillettaz and colleagues; as a result the text in the Introduction,

“However, such calibration methods are based on a discretization of the Deep model outputs into bins. Many bins are not useful as they only contain a few outputs with low values, whereas many high values fall in the same bin and are thus not discriminated. Moreover, the choice of the number of bins is left to the user, and therefore is not optimized to the Deep model nor to a specific application.”

should read:

“However, such calibration methods are based on a discretization of the Deep model outputs into bins. Many bins are not useful as they only contain a few outputs with low values, whereas many high values fall in the same bin and are thus not discriminated. Moreover, the choice of the number of bins is left to the user choice, and therefore is not optimized for the Deep Learning model nor to a specific application. Nevertheless, other algorithms propose to set a threshold by group of classes (e.g. morphologically similar clades of plankton) in order to increase the precision but at the cost of recall which falls drastically^1,2^. We present a simple, yet efficient method that accounts for uncertainty in the classifier outputs which extends previous works^1,2^ by formalizing and proposing a general framework which can be applied to control error rates with regard to ecological concerns.”
