# Supplementary material for: A new method to control error rates in automated species identification with deep learning algorithms
Source: Sci Rep. 2020 Jul 3;10:10972. doi: 10.1038/s41598-020-67573-7 (PMC7334229; doi:10.1038/s41598-020-67573-7)
Supplement: Supplementary file 1 — Supplementary file1 (DOCX 3278 kb) [file 41598_2020_67573_MOESM1_ESM.docx]

**Supplementary**
Supp. Fig. 1: The 20 reef fish species considered in the study.

| 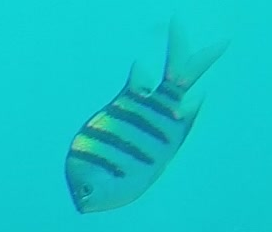 | *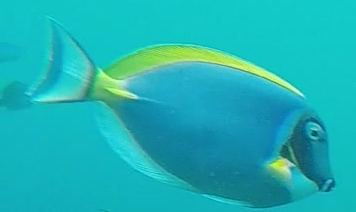* | *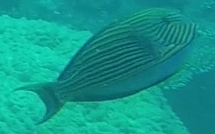* | 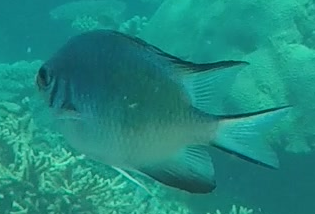 |
| --- | --- | --- | --- |
| *Abudefduf vaigiensis* | *Acanthurus leucosternon* | *Acanthurus lineatus* | *Amblyglyphidodon indicus* |
| 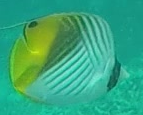 | 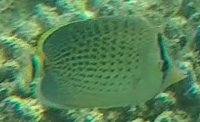 | 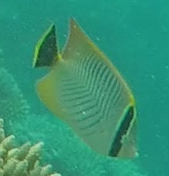 | 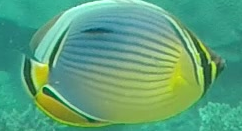 |
| *Chaetodon auriga* | *Chaetodon guttatissimus* | *Chaetodon trifascialis* | *Chaetodon trifasciatus* |
| 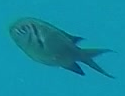 | 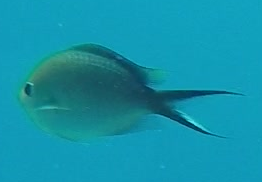 | 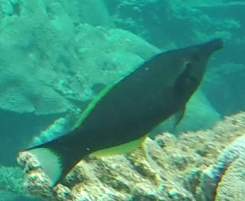 | 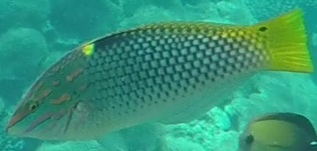 |
| *Chromis opercularis* | *Chromis ternatensis* | *Gomphosus caeruleus* | *Halichoeres hortulanus* |
| 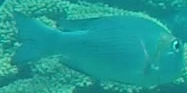 | 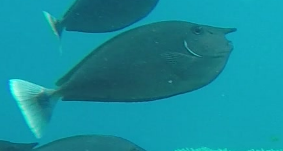 | 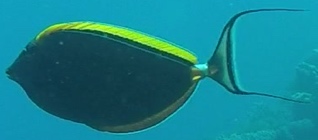 | 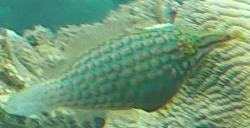 |
| *Monotaxis grandoculis* | *Naso brevirostris* | *Naso elegans* | *Oxymonacanthus longirostris* |
| *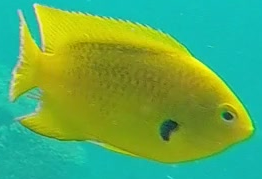* | 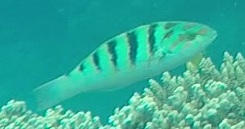 | *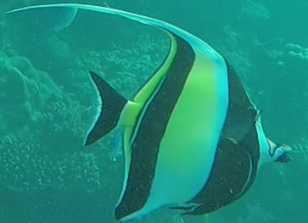* | 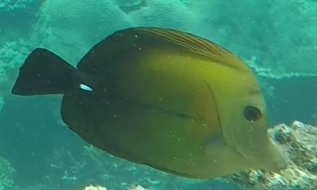 |
| *Pomacentrus sulfureus* | *Thalassoma hardwicke* | *Zanclus cornutus* | *Zebrasoma scopas* |

Supp. Fig 2: Example of training dataset augmentation

Each original image is transformed 9 times using flips and different contrast enhancements

| 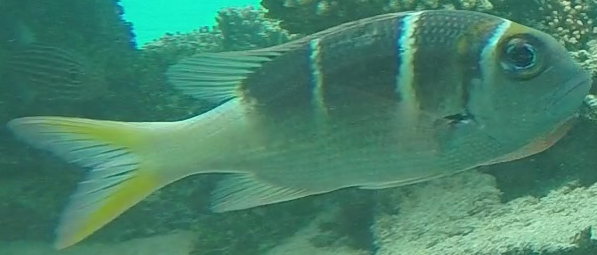 | 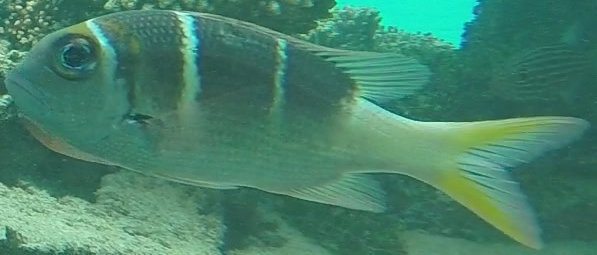 |
| --- | --- |
| Original | Original flipped |
| 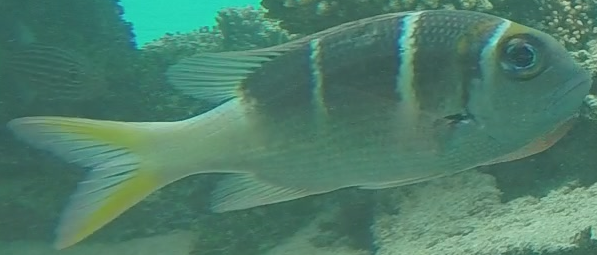 | 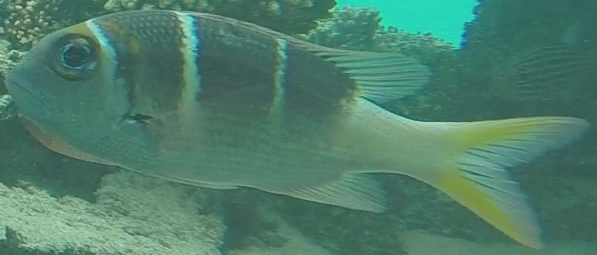 |
| Less contrast (80%) | Less contrast on flipped image (80%) |
| 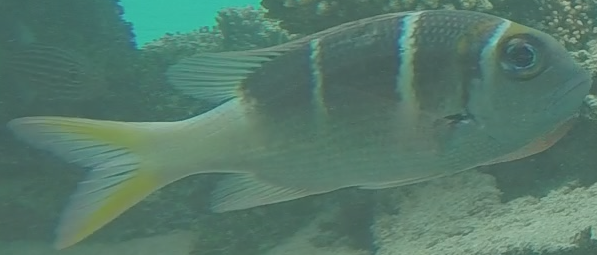 | 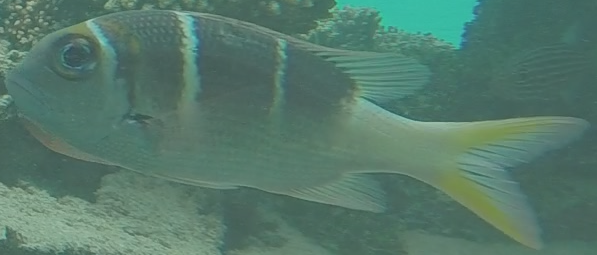 |
| Less contrast (60%) | Less contrast on flipped image (60%) |
| 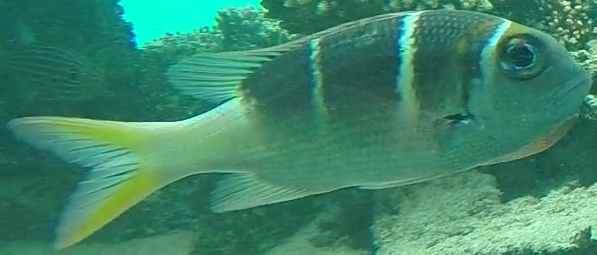 | 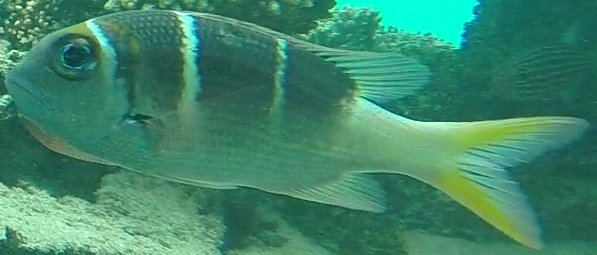 |
| More contrast (120%) | More contrast on flipped image (120%) |
| 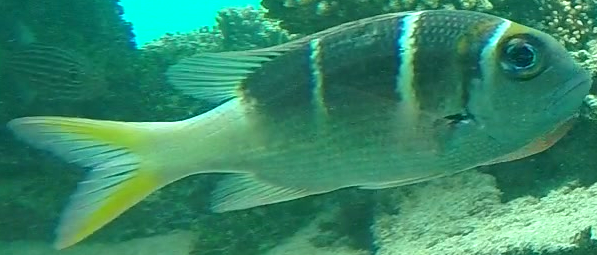 | 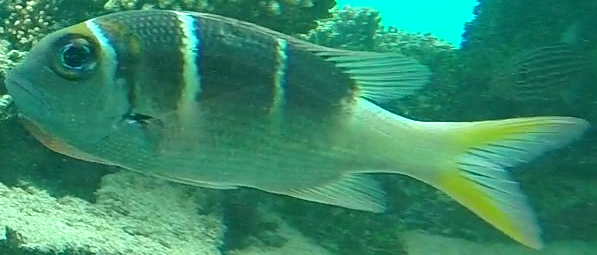 |
| More contrast (140%) | More contrast on flipped image (140%) |

Supp. Tab. 1: Number of images per species in our 3 datasets (after data augmentation).

| Family | Species | Training dataset *T0* | First dataset  *T1* | Second dataset  *T2* |  |
| --- | --- | --- | --- | --- | --- |
| Acanthuridae | *Acanthurus leucosternon* | 32,590 | 235 | 491 |  |
| Acanthuridae | *Acanthurus lineatus* | 10,080 | 114 | 864 |  |
| Acanthuridae | *Naso brevirostris* | 11,340 | 539 | 1932 |  |
| Acanthuridae | *Naso elegans* | 73,450 | 1,436 | 3,896 |  |
| Acanthuridae | *Zebrasoma scopas* | 49,700 | 48 | 579 |  |
| Chaetodontidae | *Chaetodon auriga* | 21,340 | 737 | 502 |  |
| Chaetodontidae | *Chaetodon guttatissimus* | 11,820 | 221 | 68 |  |
| Chaetodontidae | *Chaetodon trifascialis* | 52,340 | 41 | 630 |  |
| Chaetodontidae | *Chaetodon trifasciatus* | 44,210 | 71 | 82 |  |
| Labridae | *Gomphosus caeruleus* | 31,310 | 57 | 173 |  |
| Labridae | *Halichoeres hortulanus* | 31,920 | 40 | 287 |  |
| Labridae | *Thalassoma hardwicke* | 49,510 | 181 | 275 |  |
| Lethrinidae | *Monotaxis grandoculis* | 38,930 | 797 | 1,422 |  |
| Monacanthidae | *Oxymonacanthus longirostris* | 25,530 | 54 | 55 |  |
| Pomacentridae | *Abudefduf vaigiensis* | 51,240 | 376 | 216 |  |
| Pomacentridae | *Amblyglyphidodon indicus* | 11,880 | 636 | 1,310 |  |
| Pomacentridae | *Chromis opercularis* | 15,250 | 81 | 93 |  |
| Pomacentridae | *Chromis ternatensis* | 36,400 | 300 | 156 |  |
| Pomacentridae | *Pomacentrus sulfureus* | 54,090 | 270 | 142 |  |
| Zanclidae | *Zanclus cornutus* | 38,760 | 86 | 59 |  |
| TOTAL |  | 691,690 | 6,320 | 13,232 |  |

Supp. Tab. 2: Values of misclassification scores without post processing, and after processing with the threshold selected by optimizing the correct classification rate (threshold tuned and tested on the same dataset).

|  | Without post processing | Goal1 | | | |
| --- | --- | --- | --- | --- | --- |
| Species | Misclassification rate | Threshold value | Misclassification rate | | Unsure rate |
| *Chaetodon trifasciatus* | 12.19 | 94.23 | 6.10 | 6.10 | |
| *Chaetodon trifascialis* | 10 | 99.83 | 6.35 | 3.65 | |
| *Naso brevirostris* | 45.86 | 33.47 | 34.36 | 11.49 | |
| *Chaetodon guttatissimus* | 14.59 | 99.73 | 14.49 | 0 | |
| *Thalassoma hardwicke* | 9.09 | 96.15 | 1.45 | 8 | |
| *Pomacentrus sulfureus* | 9.85 | 99.66 | 2.82 | 7.04 | |
| *Oxymonacanthus longirostris* | 3.57 | 99.97 | 3.57 | 0 | |
| *Monotaxis grandoculis* | 42.89 | 40.86 | 27.78 | 15.12 | |
| *Zebrasoma scopas* | 36.96 | 66.78 | 19.17 | 19.51 | |
| *Abudefduf vaigiensis* | 0.92 | 99.71 | 0.46 | 0.46 | |
| *Amblyglyphidodon indicus* | 41.22 | 40.86 | 18.55 | 23.36 | |
| *Acanthurus lineatus* | 40.28 | 98.74 | 23.15 | 17.13 | |
| *Chromis ternatensis* | 40.38 | 33.47 | 12.18 | 31.41 | |
| *Chromis opercularis* | 38.71 | 97.52 | 19.35 | 21.50 | |
| *Gomphosus caeruleus* | 24.28 | 99.21 | 16.18 | 8.09 | |
| *Acanthurus leucosternon* | 13.85 | 96.15 | 7.94 | 5.90 | |
| *Halichoeres hortulanus* | 17.07 | 98.86 | 9.75 | 8.36 | |
| *Naso elegans* | 6.8 | 33.47 | 3.90 | 2.93 | |
| *Chaetodon auriga* | 12.95 | 99.8 | 6.37 | 6.57 | |
| *Zanclus cornutus* | 18.64 | 99.71 | 5.08 | 13.56 | |

Supp. Tab. 3: Values of misclassification scores without post processing, and after processing with the threshold selected by optimizing the Misclassification rate (threshold tuned and tested on the same dataset).

|  | Without post processing | Goal 3 | | |
| --- | --- | --- | --- | --- |
| Species | Misclassification rate | Threshold value | Misclassification rate | Unsure rate |
| *Chaetodon trifasciatus* | 12.19 | 94.22 | 0 | 12.19 |
| *Chaetodon trifascialis* | 10 | 94.63 | 3.65 | 6.35 |
| *Naso brevirostris* | 45.86 | 99.98 | 12.73 | 38.72 |
| *Chaetodon guttatissimus* | 14.59 | 99.84 | 11.59 | 7.25 |
| *Thalassoma hardwicke* | 9.09 | 99.39 | 0 | 10.55 |
| *Pomacentrus sulfureus* | 9.85 | 99.98 | 0.70 | 23.24 |
| *Oxymonacanthus longirostris* | 3.57 | 99.98 | 0 | 3.57 |
| *Monotaxis grandoculis* | 42.89 | 99.98 | 3.66 | 62.59 |
| *Zebrasoma scopas* | 36.96 | 99.9 | 1.90 | 51.64 |
| *Abudefduf vaigiensis* | 0.92 | 99.98 | 0.46 | 0.93 |
| *Amblyglyphidodon indicus* | 41.22 | 99.98 | 1.22 | 66.34 |
| *Acanthurus lineatus* | 40.28 | 99.94 | 9.26 | 32.18 |
| *Chromis ternatensis* | 40.38 | 99.98 | 0 | 75 |
| *Chromis opercularis* | 38.71 | 99.65 | 1.08 | 43.01 |
| *Gomphosus caeruleus* | 24.28 | 99.87 | 4.05 | 24.28 |
| *Acanthurus leucosternon* | 13.85 | 99.97 | 2.44 | 16.50 |
| *Halichoeres hortulanus* | 17.07 | 99.79 | 3.13 | 14.98 |
| *Naso elegans* | 6.8 | 99.98 | 0.38 | 16.30 |
| *Chaetodon auriga* | 12.95 | 99.97 | 3.39 | 13.15 |
| *Zanclus cornutus* | 18.64 | 99.95 | 0 | 25.42 |

Supp. Tab. 4: Rates of unsure, correct, and misclassifications for each goal, with a threshold learned and applied on the same dataset.

|  | Goal 1 (%) | Goal 2 (%) | Goal 3 (%) |
| --- | --- | --- | --- |
| Unsure classifications | 10.8 | 17.88 | 29.71 |
| Misclassifications | 11.19 | 6.66 | 2.07 |
| Correct classifications | 78 | 75.47 | 68.22 |

Supp. Tab. 5: For each case, the first number shows the result shown obtained with thresholds tuned in real cross validation, and the second number corresponds to the difference between benchmark conditions and real cross validation.

|  | | Goal 1 (%) | Goal 2 (%) | Goal 3 (%) |
| --- | --- | --- | --- | --- |
| Unsure classifications | 10.51 (-0.3) | | 18.80(+0.92) | 27.21(-2.5) |
| Misclassifications | | 11.95(+0.77) | 5.77(-0.89) | 2.98(+0.91) |
| Correct classifications | | 77.53(-0.46) | 75.43(-0.03) | 69.81(+1.59) |

Supp. Tab. 6: Difference between 1) results obtained with the classifier without post processing and 2) results obtained with post processing with a threshold learned on an independent dataset (cross-validation). For each case, the number shown corresponds to the results obtained with cross-validation threshold minus the results obtained without post processing*.*

|  | Goal 1 (%) | | Goal 2 (%) | Goal 3 (%) |
| --- | --- | --- | --- | --- |
| Unsure classifications | | 10.51 | 18.80 | 27.21 |
| Misclassifications | | -10.04 | -16.23 | -19.01 |
| Correct classifications | | -0.46 | -2.57 | -8.19 |

Supp. Tab. 7: Classification results of our model without post processing.

| Species | Dataset 1 (***T1***) |
| --- | --- |
| *Chaetodon trifasciatus* | 0.96 |
| *Chaetodon trifascialis* | 0.71 |
| *Naso brevirostris* | 0.45 |
| *Chaetodon guttatissimus* | 0.49 |
| *Thalassoma hardwicke* | 0.84 |
| *Pomacentrus sulfureus* | 0.90 |
| *Oxymonacanthus longirostris* | 0.87 |
| *Monotaxis grandoculis* | 0.61 |
| *Zebrasoma scopas* | 0.69 |
| *Abudefduf vaigiensis* | 0.88 |
| *Amblyglyphidodon indicus* | 0.62 |
| *Acanthurus lineatus* | 0.83 |
| *Chromis ternatensis* | 0.78 |
| *Chromis opercularis* | 0.68 |
| *Gomphosus caeruleus* | 0.72 |
| *Acanthurus leucosternon* | 0.84 |
| *Halichoeres hortulanus* | 0.92 |
| *Naso elegans* | 0.90 |
| *Chaetodon auriga* | 0.71 |
| *Zanclus cornutus* | 0.91 |
| Average | 76.33 |

Supp. Tab. 8: Rates of unsure, correct, and misclassifications for each goal. The table shows the results obtained when we tuned the thresholds on T2 and applied them on T1 (cross validation).

|  | Goal 1 (%) | Goal 2 (%) | Goal 3 (%) |
| --- | --- | --- | --- |
| Unsure classifications | 14.29 | 23.22 | 36.14 |
| Misclassifications | 11.72 | 6.36 | 1.42 |
| Correct classifications | 73.99 | 70.43 | 62.44 |
